# Aetiologies and outcomes of patients with abdominal pain presenting to an emergency department of a tertiary hospital in Tanzania: a prospective cohort study

**DOI:** 10.1186/s12876-020-01313-z

**Published:** 2020-06-05

**Authors:** Kilalo M. Mjema, Hendry R. Sawe, Irene Kulola, Amour S. Mohamed, Erasto Sylvanus, Juma A. Mfinanga, Ellen J. Weber

**Affiliations:** 1 Emergency Medicine Department, Muhimbili University of Health and Allied Science, Dar es Salaam, Tanzania; 2 Emergency Medicine Department, Muhimbili National Hospital, Dar es Salaam, Tanzania; 3 Department of Emergency Medicine, University of California, San Francisco, CA USA

**Keywords:** Abdominal pain, Non-traumatic patients, Emergency department, Tanzania, Sub Saharan Africa

## Abstract

**Background:**

Abdominal pain in adults represents a wide range of illnesses, often warranting immediate intervention. This study is to fill the gap in the knowledge about incidence, presentation, causes and mortality from abdominal pain in an established emergency department of a tertiary hospital in Tanzania.

**Methods:**

This was a prospective cohort study of adult (age ≥ 18 years) patients presenting to the Emergency Medicine Department of Muhimbili National Hospital (EMD-MNH) in Dar Es Salaam, Tanzania with non-traumatic abdominal pain from September 2017 to October 2017. A case report form was used to record data on demographics, clinical presentation, management, diagnosis, outcomes and patient follow-up. The primary outcome of mortality was summarized using descriptive statistics; secondary outcome was, risks for mortality.

**Results:**

Among 3381 adult patients present during the study period, 288 (8.5%) presented with abdominal pain, and of these 199 (69%) patients were enrolled in our study. Median age was 47 years (IQR 35–60 years), 126 (63%) were female, and 118 (59%) were referred from another hospital. Most common final diagnoses were malignancies 71 (36%), intestinal obstruction 11 (6%) and peptic ulcer disease 9 (5%). Most common EMD interventions given were intravenous fluids 57 (21%), analgesia 49 (25%) and antibiotics 40 (20%). 160 (80%) were admitted of which 15 (8%) underwent surgery directly from EMD. 24-h and 7-day mortality were 4 (2%) and 7 (4%) respectively, while overall in hospital-mortality was 16 (8%). Among the risk factors for mortality were male sex Relative Risk (RR) 2.88 (*p* = 0.03), hypoglycemia (RR) 5.7 (*p* = 0.004), ICU admission (RR) 14 (*p* < 0.0001), receipt of IV fluids (RR) 3.2 (*p* = 0.0151) and need for surgery (RR) 6.6 (*p* = 0.0001).

**Conclusion:**

Abdominal pain was associated with significant morbidity and mortality as evidenced by a very high admission rate, need for surgical intervention and a high in-hospital mortality rate. Future studies and quality improvement efforts should focus on identifying why such differences exist and how to reduce the mortality.

## Background

Abdominal pain is one of the most important and challenging symptoms that brings a patient to the physician for evaluation. Abdominal pain represents a spectrum of diseases ranging from the most benign and self-limited to surgical emergencies [1, 2]. Abdominal pain is one of the most common presentations to the emergency department. Studies from high income countries suggest that abdominal pain presentation at the ED has an incidence of 7–10% [3].

In general, only a quarter of the patients with abdominal pain need surgical interventions, in such cases the dilemma remains whether surgery is needed emergently [4, 5]. About 35–41% patients with abdominal pain are admitted while a quarter of the patients are discharged [3]. Even with modern diagnostic tools and improved surgical skills older age and comorbid conditions pose a relatively higher morbidity and mortality [6]. Increased risk is found in populations with diabetes and those who are immunocompromised, children and the elderly; there is six to eight-fold increase in the mortality in the elderly compared to younger patients [7].

Most information about abdominal pain emergencies comes from High Income Country (HIC) where the most common aetiology is non-specific abdominal pain even after all the appropriate laboratory and imaging investigations, this provides little guidance to the patients in our setting where majority cannot afford all investigations [8]. It is best to base care with findings from Low Income Country (LIC) due to differences in geographical distribution, cultural practices and health care systems.

Information about abdominal pain emergencies in low and middle-income countries is limited.

Lack of documented clinical profiles, presentations and outcomes poses a challenge to creating and meeting the standards of care. This study is to fill the gap in the knowledge about incidence, presentation, causes and mortality from abdominal pain in an established emergency department of a tertiary hospital in Tanzania.

## Methods

### Study design

This was a prospective cohort study of all non-traumatic adult patients presenting to the EMD MNH with abdominal pain for five weeks, from 4^th^ September 2017 to 10^th^ October 2017.

### Study setting

This study was conducted at the EMD- MNH, Dar es Salaam, which is situated in Ilala, one of the five districts of Dar es Salaam - Tanzania. MNH is the only tertiary teaching hospital which serves as a National referral hospital with a bed capacity of 1500 beds with weekly admissions of around 1000 to 1200 patients [9]. The EMD-MNH was inaugurated in 2010 and it receives all emergency referral cases from hospitals all over the country. The EMD sees more than 200 patients on a daily basis including all populations ages except neonates.

### Study participants

All consenting adults with age greater than or equal to 18 years presenting with abdominal pain unrelated to a recent trauma were eligible for the study. We excluded patients that developed cardiac arrest while in ED before being enrolled, those needing immediate resuscitation and those that discharged themselves against medical advice.

### Study protocol

A research assistant was scheduled to collect data of consenting study participants that met inclusion criteria over 12 h, either during the day 0800-2000 h or night 2000-0800 h. This was done in the course of 5 weeks of the study at the convenience of the presence of the research assistant. Demographics, clinical presentation, initial management, and outcomes were documented both from the patient/care giver through interview and hospital Electronic Medical record system named WELLSOFT for all enrolled patients after written consent. A structured case report form then used to record all participants’ information. All patients were followed up in a hospital ward (if admitted) or through mobile phone calls to determine their outcome from the ED-MNH, at 24-h and 7-days. This was made possible by having patients and relative’s phone number documented in the data collection tool and calls were made on the specified time to see how the patient was doing.

### Outcomes

The primary outcome was mortality and secondary outcome was risk factors for mortality.

### Data analysis

Information was inputted into REDCap (version 7.2.2, Vanderbilt, Nashville, TN, USA) and transferred into the Statistical Package for Social Science (SPSS) (version 22.0.0, IBM, LTD, North Carolina, USA). Descriptive statistics are reported with mean and standard deviation for normally distributed data while median and interquartile range were calculated for non-parametric data. Proportions was used to describe incidence of adult patients presenting with non-traumatic abdominal pain at the EMD, during the study period and for categorical descriptive variables. Univariate relative risk with 95% confidence intervals was used to determine predictors of mortality, *P* values of < 0.05 were considered statistically significant.

## Results

**Fig. 1:**
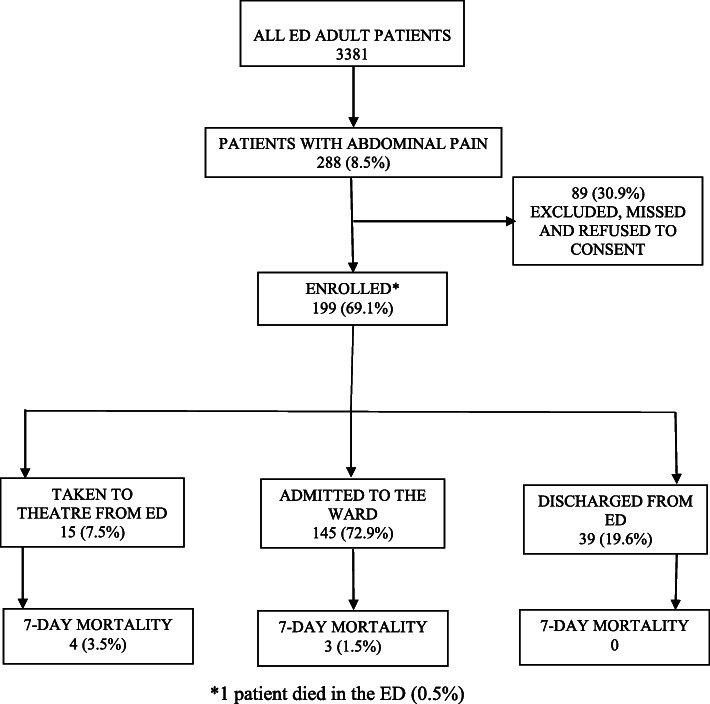
Study Flow Diagram

A total of 3381 adult patients presented to the ED during the study period and 288 (8.5%) presented with the complaint of abdominal pain. 89 patients were missed (research assistant not present), did not meet inclusion criteria or refused to consent. In total, 199 (69%) consented to participate in the study. There was no loss to follow up. (Fig. 1).

### Demographics and clinical profiles

Of the 199 patients enrolled, 126 (63%) were female and median age was 47 years (IQR 35–60). Most of the patients (118, 59%) were referred from other hospitals. There was a previous history of surgery in 37 (19%), hypertension in 30 (15%) and known malignancy in 18 (9%). (Table 1) Associated symptoms commonly reported by patients included abdominal distension 46 (23%), vomiting 38 (19%) and constipation 25 (13%). 37% presented with tachycardia and 9% with tachypnea. Fever, hypothermia, and hypotension were rare. On examination, abdominal tenderness was present in 119 (60%) and distension in 67 (34%). 15% had a palpable mass. (Table 2).

**Table 1:** Demographic characteristics of adult patients presenting with abdominal pain

Variable	Total ***N*** = 199	Alive ***N*** = 178	Dead ***N*** = 21
	**n (%)**	**n (%)**	**n (%)**
**Age in years in groups**			
18–35	52 (26.1)	42 (80.8)	10 (19.2)
36–45	43 (21.6)	41 (95.3)	2 (4.7)
46–65	75 (37.7)	69 (92)	6 (8)
> 65	29 (14.6)	26 (89.7)	3 (10.3)
**Gender**			
Male	73 (36.7)	63 (86.3)	10 (13.7)
Female	126 (63.3)	120 (95.2)	6 (4.8)
**Referral status**			
Self-referral	81 (40.7)	76 (93.8)	5 (6.2)
Referred	118 (59.3)	107 (90.7)	11 (9.3)
**Past Medical History**			
Previous history of surgery	37 (18.6)	34 (91.9)	3 (8.1)
Hypertension	30 (15.1)	26 (86.7)	4 (13.3)
Known malignancy	18 (9.0)	15 (83.3)	3 (16.7)
Diabetes	8 (4.0)	7 (87.5)	1 (12.5)
HIV	8 (4.0)	7 (87.5)	1 (12.5)

**Table 2:** Associated symptoms and physical findings on abdominal examination

Variable	Frequency N	Percentage	
**Clinical presentation**			
Abdominal distension	46	23.1	
Vomiting	38	19.1	
Constipation	25	12.6	
Diarrhoea	9	4.5	
Fever	9	4.5	
**Physical findings**			
Tenderness	119	59.8	
Distension	67	33.7	
Normal	46	23.1	
Palpable mass	29	14.6	
Guarding	6	3.0	

### Emergency provider diagnosis and final diagnosis

The most frequent ED provider diagnoses were malignancy 67 (34%), intestinal obstruction 15 (8%) and Upper gastrointestinal bleeding 10 (5%) while final hospital diagnosis included malignancy 71 (36%), intestinal obstruction 11 (6%) and PUD 9 (5%). (Table 3) 39 (20%) patients were not admitted, of those admitted, 70 patients (44%) were admitted to the surgical ward, 48 (30%) to medical ward,40 (25%) to the OBGYN ward and 2 (1%) to the ICU.

**Table 3:** Top 10 diagnosis

Variable	Total done N = 199	Alive	Dead
**Top 10 Final Diagnosis**	***n (%)***	***n/N (%)***	***n/N (%)***
Malignancy	71 (35.7)	55/71 (77.5)	6/71 (22.5)
Intestinal obstruction	11 (5.5)	6/11 (54.5)	5/11 (45.5)
Peptic ulcer disease	9 (4.5)	9/9 (100)	0
Benign prostatic hypertrophy	7 (3.5)	7/7 (100)	0
Chronic kidney disease	7 (3.5)	6/7 (85.7)	1/7 (14.3)
Upper GI bleeding	6 (3.0)	5/6 (83.3)	1/6 (16.7)
Incomplete abortion	6 (3.0)	6/6 (100)	0
Peritonitis	6 (3.0)	4/6 (66.7)	2/6 (33.3)
Gastritis	6 (3.0)	6/6 (100)	0
Pelvic inflammatory disease	5 (2.5)	5/5 (100)	0

### Risk factors associated with mortality

24-h and 7-day mortality were 4 (2%) and 7 (4%) respectively, while overall in hospital-mortality was 16 (8%). In relative risk analysis factors significantly associated with in-hospital mortality were being a male patient RR 2.9, tachypnea with RR > 22 cpm RR 3.4 and requirement for ICU admission RR 14.1. (Table 4).

**Table 4:** Risk factors associated with mortality

Variable	Total N (%)	Died n (%)	Relative risk 95% CI	***p***-value
Age > 65	29 (14.6)	3 (10.3)	1.4 (0.4–4.5)	0.6
Male	73 (36.7)	10 (13.7)	2.9 (1.1–7.6)	0.03
Referred	118 (59.3)	11 (9.3)	1.5 (0.5–4.2)	0.4
**Past medical history**				
Previous surgery	37 (18.6)	3 (8.1)	0.1 (0.03–0.4)	0.0003
Malignancy	18 (9.0)	3 (16.7)	2.3 (0.7–7.4)	0.1
Diabetes	8 (4.0)	1 (12.5)	1.6 (0.2–10.6)	0.6
HIV	8 (4.0)	1 (12.5)	1.6 (0.2–10.6)	0.6
**Abnormal vitals**				
MAP< 65 mmHg	5 (2.5)	1 (20)	2.6 (0.4–16)	0.3
HR > 100 bpm	74 (37.2)	8 (10.8)	2.2 (0.8–6.1)	0.1
RR > 22 cpm	18 (9.0)	4 (22.2)	3.4 (1.2–9.3)	0.02
HDU/ICU admission	2 (1.0)	2 (100)	14.1 (8.5–23.3)	< 0.0001

## Discussion

To the best of our knowledge, this is the first study to highlight the burden of acute abdominal pain within the EMD in East Africa. We found that 8.5% of the adult patients who presented to the EMD-MNH had abdominal pain. There is a lack of previous studies in Tanzania for patients with abdominal pain; the overall burden of abdominal pain and most common diagnoses had not been studied. The proportion we found in this study is within the same range as HICs, which have reported a incidence of 7–10% [10].

Most of these patients commonly had associated symptoms such as abdominal distension, vomiting and constipation of which are the classical hallmarks in patients with intestinal obstruction amongst others. The findings are somewhat similar in studies in HICs where these symptoms commonly observed were vomiting anorexia and fever [11].

In our study, females presented twice as frequently as males with abdominal pain; this is different from studies in HICs which show male predominance [11]. This may reflect the fact that within the culture of Tanzania, females are more likely to seek health care than males.

The most frequent specific EMD and hospital diagnoses in our study was intra-abdominal malignancy followed by intestinal obstruction. This finding is in contrast to similar studies done in Nigeria and Kenya where surgical emergencies such as appendicitis and ectopic pregnancy were commonly found, while intra-abdominal malignancies were much rarer [12–14]. In HIC there was almost similar findings to those done in Kenya and Nigeria with respect to aetiology.

Furthermore, the findings in our patient population may be due to the fact that many are referred from outlying hospitals for specialized surgical and oncologic services. These referring hospitals have the capability of surgical intervention and therefore may be able to handle the more common presentations such as appendicitis and hernias. However, there is no routine screening for malignancies in Tanzania, and most of these patients present rather late, and thus these patients are more likely to be referred to a tertiary hospital. This finding emphasizes the need for strengthening preventive services and surgical services at the municipal levels so that these patients receive surgical interventions as early as possible.

Two thirds of the patients with abdominal pain were admitted after evaluation at the EMD highlighting the acuity of illness in our cohort. This high acuity is also reflected in the in -hospital mortality rate of 8% compared to HIC of less than 1% and those that needed ICU admission had an increased risk factor to mortality. In our study, two thirds of patients with abdominal pain were referred from peripheral hospitals; the referring hospitals commonly were the municipal hospitals within Dar es salaam.

The observed factors that were associated with in-hospital mortality in our cohort were male patients, hypoglycemia with RBG < 3 mmol/L, tachypnea on presentation, and need for surgery.

The mortality rate increased significantly to 8% as a final outcome while that at 7 days was 4%.

### Limitations

This was a single center study, however EMD-MNH receives referral from all over the country every day and the research assistant made sure to capture all patients that met the inclusion criteria during the randomly selected 12 h of the day that she was present for data collection. However, it is possible that the frequency of the diagnoses of patients referred are not typical of the country as a whole.

Relying on the informant’s report in acutely ill patients might have resulted in lack of complete data. This was mitigated by a careful history from the patient or informant present with the most details and physical examination done by the provider while evaluating the patient. Investigations were ordered at the discretion of the physician, and thus not all patients received all tests.

## Conclusion

Abdominal pain is a common complaint amongst adult patients presenting to the EMD-MNH. The most common aetiologies and outcomes are different from HIC, with patients in our setting having higher acuity and higher mortality. Future studies and quality improvement efforts should focus in identifying why such differences exist and how to reduce the mortality.

## Data Availability

The dataset supporting the conclusion of this article is available from the authors on request.

## Abbreviations

AIDS: Acquired Immune Deficiency Syndrome
CT KUB: Computed Tomography Kidney-Ureter-Bladder
CT SCAN: Computed Tomography Scan
GBD: Global Burden of Diseases
IRB: Institutional Review Board
LIC: Low Income Countries
MNH: Muhimbili National Hospital
MUHAS: Muhimbili University of Health and Allied Sciences
POC: Point of Care
PUD: Peptic Ulcer Disease
